# The nutrition and therapeutic potential of millets: an updated narrative review

**DOI:** 10.3389/fnut.2024.1346869

**Published:** 2024-04-30

**Authors:** Jinu Jacob, Veda Krishnan, Chris Antony, Masimukka Bhavyasri, C. Aruna, Kiran Mishra, Thirunavukkarasu Nepolean, Chellapilla Tara Satyavathi, Kurella B. R. S. Visarada

**Affiliations:** ^1^ICAR-Indian Institute of Millets Research, Hyderabad, India; ^2^Division of Biochemistry, ICAR-Indian Agriculture Research Institute, New Delhi, India; ^3^National Institute of Indian Medical Heritage, Hyderabad, India; ^4^SIES College of Arts, Science and Commerce, University of Mumbai, Mumbai, India

**Keywords:** ancient grains, nutricereals, life style disorders, malnutrition, micronutrients, cancer, celiac disease, vitamins

## Abstract

Millets are ancient small grains grown in arid and semiarid regions of the world. They are staple food for many people in Asia and Africa. They are abundant sources of minerals and vitamins, giving them the name Nutricereals. Moreover, millets contain valuable phytochemicals that impart therapeutic properties for various disorders and diseases, thus giving them nutraceutical value. A wide array of biochemical compounds are present in the plant parts as well as the grains. In the oldest texts of medicine in India and China, millets are mentioned for use for their medicinal value. There has been expanding interest and emerging facts about millets and their therapeutic uses. Ample evidence shows that consumption of millets amounts to correction of life style and metabolic disorders. Therapeutic properties of millets can be viewed in two ways, supplementary nutrition through minerals and vitamins, and therapeutic value through the presence of phytochemicals and specialty compounds that include flavonoids, phenolics, anthocyanidins and others that have antioxidant potential. Millets are gluten free, have low glycemic index and the phytochemicals aid in correction of lifestyle disorders and prevention of ailments like carcinogenesis. Supplementary benefits include treatment of anemia and calcium deficiency especially for pregnant women and young children. With the improvements in analytical methods for detection of various compounds, it is possible to identify the compound-specific genotypes in millets that can cater to the pharmacy industry. End-use specific genotypes can be bred to meet the demand. Millets being climate resilient, can contribute to a healthier life and better world through economic usage of natural resources.

## 1 Introduction

**FIGURE 1:**
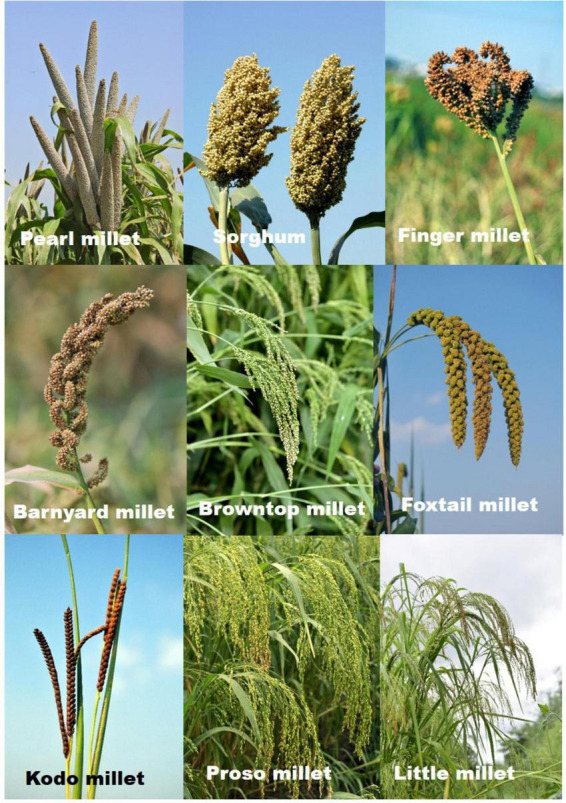
Panicles of different millets.

Millets are small-grained multipurpose cereals and impart medicinal value by virtue of the presence of vitamins, minerals, and bioactive compounds that aid in the recovery and well-being of human health. Due to their high nutritional value, these archeological staples are also called nutricereals. Millets comprise major millets (sorghum, pearl millet, and finger millet) and minor millets (foxtail millet, little millet, kodo millet, proso millet, brown top millet, fonio, teff, and barnyard millet) (Figure 1). Major millets can be used directly after harvest and cleaning, while the minor millets need primary processing for gentle removal of the outer layers of grain that also contain many antioxidants. There has been a steep increase in awareness of the nutritional benefits of millets, thus increasing the demand for millets as food and value-added products (1). Millets contain about 65–75% carbohydrates, 7–12% proteins, 2–5% fat, and 8–15% fiber. They have a higher content of essential amino acids compared to conventional cereals and prolamin in millets increases the digestibility of proteins. While the nutritional advantages of millets have been realized through knowledge sharing in recent times, the therapeutic value of millets needs attention. There is robust scientific evidence to suggest that consumption of millets reduces the progression of prediabetes, results in better glycemic control, reduces body mass index (BMI), and mitigates atherosclerotic cardiovascular disease risk (2). High resistant starch (RS) and slowly digestible starch (SDS) in millets, causes lower postprandial glucose and insulin excursions (3). Millets are gluten free and are the choice diet for celiac patients. Due to their nutritional value millets are picking up market for weaning as well as conscious foods. Phytochemicals like proanthocyanidins present in the grain and bran of the millets possess anti-obesity effects by inducing satiety. Millet foods and products for health, prevention and therapy are known from ancient times, yet are to be expanded worldwide. In this review, we enumerate the different medicinal and therapeutic properties of millets in preventing and curing the lifestyle disorders, specific diseases and ailments along with the history of millets in traditional medicine.

## 2 Millets in ancient texts and scriptures and traditional medicine

The history of millets dates to the history of the grasses and humanity even before man began his settled life and he was a hunter-gatherer. Research reveals that millets were consumed in the Indus valley civilization, flourishing from 3,300 to 1,300 BC. Millets were cultivated as summer crops along with rice and tropical pulses at early settlements inside and outside the Indus zone, which is the evidence for millet consumption in the pre-Vedic period (before 1700 BC) (4). The Harappans used millets and being drought resistant, they were cultivated in the peripheral region due to the fall in monsoons. Finger millet, sorghum, pearl millet along with little millet and foxtail millet, were found to dominate the cultivation during the mature and late Harappan period (5, 6). Their studies prove that early inhabitants of the Gangetic plain from the 3rd millennium BC to the 2nd century BC cultivated rice, wheat, and African and indigenous millet varieties. Vedas are considered the oldest repositories of knowledge, and millets are mentioned in various instances (7). The Indian scriptures, the *Puranas*, also mention the names of various wild and cultivated millets used for rituals and as food and fodder (8). The use of millets like *thinai* (foxtail millet) was famous in many dishes during the Chola era (9*^th^* century). Sorghum is used in the marriage rituals of Telangana state of India till today.

Ayurveda is the ancient science of medicine in India and a variety of millets are mentioned, and their qualities are detailed in all treatises of the *Ayurvedic* system of medicine, which flourished during the *Samhita* period (450 BC- 400 AD) and *Sangraha* period (400–700 AD). As per Charaka Samhita, a prominent treatise of *Ayurveda*, millets are said to be *kashaya* (astringent) and *madhura* (sweet) in taste, light in digestion, aggravating *vāta*, and pacifying *kapha* and *pitta*. Many Nighantus (encyclopedic lexicons) in *Ayurveda* have referred to millets as sweet and astringent in taste but pungent in effect after digestion. The yellow variety of foxtail millet is described as the best among millets in the *Ayurvedic* writings. It is also considered good for fracture healing and stoutening (9). Repeatedly, *kodrava* (kodo millet) is mentioned as *visahara* (alleviating poison) by Kaiyadeva Nighantu and Dhanvantari Nighantu. It is also advised to be good for healing ulcers. However, the excessive consumption of *kodrava* (kodo millet) and *uddālaka (vanakodrava*, wild Kodo millet*)* is said to cause *raktapitta* (bleeding disorders) in *Garuda Purana* (10).

In China, millets are considered a sacred crop. The leader of the Shang Dynasty in the 2*^nd^* millennium BC was known as Hou Chi ‘The ruler of Millet.’ In traditional Chinese medicine, millet is mentioned as cooling and diuretic, strengthens kidney energy and builds yin fluids, moistens dryness. Thus, making them perfect food to consume during the summer months, and in preparation for the autumn. Archaeobotanical analysis of plant remains found in five pottery model granaries in burial sites in China proves that foxtail and common (proso) millet were cultivated on a larger scale than wheat or other cereals. Millet-based multi-crop farming dominated China’s regional agricultural system during the Western Han Dynasty (202 BC-AD 8) (11). Foxtail millet (*Setaria italica*) and broomcorn millet (*Panicum miliaceum*) were traditionally the most important cereals cultivated in North China and were first identified at 7,000-year-old Neolithic sites (12).

Millets have traveled throughout the Middle East and Northern Africa, where it became a staple and typical food of the Sumerian diet at about 2500 BC. The Hebrew bible makes mention of humble millet. The Hanging Gardens of Babylon were said to have included millet among their treasured plants. It was pointed out in the Bible that millets were used to prepare bread. *Panicum colonum* (a wild ancestor of *Sawa* millet, barnyard millet), was found in the Egyptian tombs of mummies of the Junstein Age (13).

## 3 Nutritional profile of millets and their potential as nutrient supplementation

Millets stand out among cereals due to their high levels of dietary fiber, antioxidants, and proteins. Millet grains primarily consist of carbohydrates with varying amounts of proteins, fats, and dietary fiber (Table 1). Carbohydrates are the largest fraction of the total grain weight, with variations among different millet types (14). Starch is the major carbohydrate in the grains and controls millets digestion and glycemic response. Grain starch consists of amylose and amylopectin along with dietary fibers and meager levels of free sugars like glucose and sucrose (15). Protein content ranges from 6 to 13%, proso millet and foxtail millet being the richest sources (Table 1). Millet proteins have a relatively high content of essential amino acids such as lysine, methionine, and cysteine, which are limited in other cereal grains like rice and wheat (16). Millet grains contain moderate levels of fats, typically ranging from 2 to 8% of the grain weight, pearl millet being the richest. Millet fats are composed of unsaturated fatty acids such as linoleic acid and oleic acid, contributing to nutritional value and health benefits (17, 18). Quantity of dietary fiber in millet is almost double that in rice and comparable to whole wheat. Dietary fiber in millets consists of both soluble and insoluble fractions (Table 2); the soluble fibers, including β-glucans, arabinoxylans, and pectins; and insoluble fibers, such as cellulose and hemicellulose (19). Pearl millet and finger millet are prominent sources of dietary fiber. Millets are also rich in micronutrients like calcium (10–348 mg/100 g), iron (2.2–17.7 mg/100 g), zinc (0.4 –2.8 mg/100 g), and phosphorus (189–293 mg/100 g), vitamins such as thiamine (0.15–0.60 mg/100 g), niacin (0.89–4.6 mg/100 g), and riboflavin (0.9–0.28 mg/100 g) (Table 1) (16, 20–22). Pearl millet has the highest iron content of 5–6.5 mg/100g. Finger millet is one of the richest vegetarian sources of Calcium (300–350 mg/100g), which is almost 10 times that in wheat. Secondary metabolites in millets are phenolics (phenolic acids, flavonoids, and tannins), phytosterols, and policosanols that serve as antioxidants and minimize free radical damage to the body. The B group of vitamins, thiamine and riboflavin, are rich in millets compared to wheat and rice (Table 1). Sorghum grain is a rich source of micro and macro nutrients offering potential health and therapeutic benefits (23).

**TABLE 1:** Nutritional composition of 8 different types of millets in comparison to rice and wheat/100 gm.

Nutrient	Finger Millet	Foxtail Millet	Pearl Millet	Barnyard Millet	Kodo Millet	Little Millet	Proso Millet	Brown Top Millet	Rice	Wheat
Protein (g)	7.3	12.3	11.6	6.2	8.3	7.7	12.5	8.98	6.4	11.8
Fiber (g)	3.6	8	1.3	10.1	9.3	7.6	2.5	7.3	0.3	2
Calcium (mg)	344	31	42	20	27	17	14	28	2	29
Iron (mg)	3.9	1.1	2.8	15	0.6	9.3	3.8	7.72	0.6	3.9
Zinc (mg)	2.8	1.1	1.7	0.4	1.2	1.2	1.7	2.5	0.9	2.7
Phosphorus (mg)	283	258	285	293	189	207	277	276	52	288
Thiamine (mg)	0.42	0.59	0.27	0.33	0.15	0.3	0.41	–	0.4	0.24
Niacin (mg)	1.1	3.2	0.89	4.2	2.1	3.2	4.6	–	1.6	5.5
Riboflavin (mg)	0.19	0.11	0.15	0.11	0.09	0.09	0.28	–	0.03	0.13

The nutritional values will change depending on the growth climate and the species being used (16, 20–22).

**TABLE 2:** Dietary Fiber composition of eight different types of millets.

Millet variety	Fiber content (g/100g)	Fiber type and composition (approximate g/100g)		References
		Soluble DF*	Insoluble DF **	
Finger Millet	15.00	1.40	15.70	(52)
Foxtail Millet	8.00	5.87	11.06	(140)
Pearl Millet	8.00	1.91	9.85	(141)
Barnyard Millet	6.00	4.15	8.19	(142)
Kodo Millet	9.00	3.85	13.13	(52)
Little Millet	7.00	5.65	8.57	(141)
Proso Millet	3.00	2.05	9.48	(141)
Brown Top Millet	9.00	–	–	(21)

The nutritional values will change depending on the growth climate and the species being used.

*Mainly β-glucan and arabinoxylan.

**Lignin, cellulose, hemicelluloses, water-unextractable arabinoxylan.

Consumption of millets helps in preventing the metabolic disorders and in correction of life style disorders. Since these are administered as food it becomes easy for consumption and the bioavailability will be more. Diet based trials with millet supplementation have shown encouraging results on health and performance. Trials on supplementation of diet with millets have been promising, showing improvement in health and performance including anemia (24). Regular supplementation of multi-millet health mix (kodo millet, little millet, foxtail millet, finger millet, and wheat with the inclusion of pulses) to primary school children in India showed a positive effect in increasing the anthropometric indices (25). Khader and Maheswari (26) found that there was significant increase in weight of preschool children after supplementation of amylase-rich malted millet mixes for the period of 4 months. A randomized clinical trial program through food based approach using pearl millet *ladoo* (Indian sweet) showed a significant rise in mean hemoglobin (Hb) levels of adolescent girls (27). Dietary supplementation of adolescent school girls with finger millet porridge improved hemoglobin levels (28).

## 4 Role of millets in correcting diseases and lifestyle disorders

Malnutrition prevails in many countries and estimates by WHO indicate that 22.3% of all children under 5 years globally are stunted (29). At the same time, obesity and NCDs (non-communicable diseases) are on the rise. As per WHO, NCDs kill around 41 million people globally of which cardiovascular diseases account for the maximum number of deaths (17.9 million) followed by cancers (9.3 million) and diabetes (2 million) (30). About 16% of adults aged 18 years and older worldwide are obese and the worldwide prevalence of obesity in adults as well as children has more than doubled over the last three decades (31). Among NCDs diabetes, hypertension, cardiovascular diseases and cancer stand at the top, the others include gut health impairment, obesity, thyroid dysfunction etc. As per WHO estimates NCDs are responsible for almost 74% of all deaths globally.

### 4.1 Effect of millets on type-2 diabetes mellitus (T2DM)

Diabetes is a worldwide epidemic and the disease is characterized by rising levels of blood sugar content due to reduced insulin action or an absolute lack of it. Millets are ideal food for regulating diabetes and the underlying mechanisms have been reported by many. Hypoglycemic effect of millets is associated with proportion of low digestible starch fractions (3), potency of phenolics in limiting carbohydrate digestion (32), ability to reduce the reactive oxygen species (ROS), increase in the abundance of probiotic bacteria, activation and /or inhibition enzymes and regulate various signaling pathways (33–35). Phenolic compounds in millets regulate the oxidative stress in the cells and protect the pancreatic β cells. β-glucan isolated from *Eleusine coracana* seeds, Ec-βG was found to be an active inhibitor for α- amylase and α-glucosidase that demonstrated antidiabetic activity (36). Phenolics from sorghum and finger millet have inhibitory effects on starch-digesting enzymes like salivary and pancreatic α-amylases and α-glucosidase, which effectively reduce post-prandial blood glucose levels (37–39). Polyphenol-enriched extract from pearl millet (*Pennisetum glaucum*) inhibits key enzymes involved in postprandial hyperglycemia (α-amylase, α-glucosidase) and regulates hepatic glucose uptake (32). Phenolic compounds impart antiglycation activities and prevent the formation of advanced glycation end (AGE) products. *p*-coumaric and chlorogenic acids found abundantly in barnyard millet caused a significant decrease in advanced glycation end-products and protected against glycoxidation-induced protein conformational changes (40).

Detailed studies in rat and mice models show that feeding diabetic rats with kodo and finger millet-based diets reduced blood glucose levels (41). Glycemic and oxidative stress was reduced as indicated by low levels of lipid peroxidation in millet-fed rats when compared to control rats through the inhibition of glycation of tail-end collagen. Hypoglycemic effect of ethanolic extract of sorghum and foxtail millet in diabetic rats was *via* inhibition of hepatic gluconeogenesis and was like anti-diabetic medication (42, 43). Supplementation of millet-based diet preparations lowered glycemic response in diabetic rat models mainly owing to the i) high dietary fiber content (2), ii) presence of peptides (44) and iii) starch (45). Foxtail millet starch and protein components alleviated impaired glucose tolerance and abnormal lipid contents and improved glucose metabolism in diabetic rats (46). Japanese barnyard millet protein showed ameliorative activity in diabetic mice (47). Barnyard millet bran-based diet reduced diabetic polyuria, water intake, and HbA1c levels in diabetic rats (48).

Foxtail millet prolamin supplementation improved glucose intolerance and insulin resistance in diabetic mice, improved liver function impairment and modulated serum metabolic profiles, especially of retinol and riboflavin metabolism (49). Whole grain proso millet diet improved glucose tolerance, liver and kidney injury, and insulin resistance in diabetic mice through modulating the PI3K/AKT signaling pathway through miRNA regulation (50). Protein isolates from raw and cooked foxtail millet improved and altered diabetes-induced gut dysbiosis (51). Further *in vitro* studies indicated that prolamins from foxtail millet exhibited α-amylase inhibiting properties in raw as well as cooked form and cooked prolamins had a superior effect (44). Millet dietary fiber reduces glycemic reactions through changed lipid metabolism, altered bile acid metabolism, and glucose level improvement (52).

Millets were effective in curing diabetes-associated complications such as cataracts, dermal wounds, fatty liver, etc. Finger millet-fed diabetic rats exhibited hastened dermal wound healing (53). through altered activities of antioxidant enzymes, enhanced expression of nerve growth factor (NGF), increased synthesis of collagen, and activation of fibroblasts and mast cells. Finger millet seed phenolics delayed cataract genesis in diabetic rats by lowering the activity of lens aldose reductase (AR), serum advanced glycation end products and blood glycosylated Hb levels (39, 54).

Starch and protein components in foxtail millet significantly increased *Lactobacillus* species, reduced gut microbiota dysbiosis caused by diabetes, thus alleviating hyperglycemia, and liver dysfunction in diabetic mice. Foxtail millet alleviated non-alcoholic fatty liver disease (NAFLD)-related gut microbiota dysgenesis, a diabetes-associated complication, in a mice model fed on a high-fat diet (46). By putting genetically type 2 diabetic KK-Ay mice on a Korean foxtail millet protein diet, insulin levels decreased greatly, and insulin sensitivity was improved through adiponectin intervention (55).

Clinical trials indicated that increased consumption of foxtail millet reduced mean fasting blood glucose levels and mean 2 h-glucose levels. There was a significant increase in blood leptin (‘leptin’ is a key appetite-regulating hormone that normalizes hyperglycemia), insulin resistance reduction, and marginal reduction in inflammation (33). Increasing the intake of a millet-based diet in patients with T2DM improved glycemic control, decreased hyperinsulinemia, and lowered plasma lipid concentrations (56). Consumption of fermented and germinated foxtail millet alleviated diabetic kidney disease, another complication of prolonged diabetes (57). Regular consumption of millets by humans translates into better post-prandial blood glucose and better HbA1c levels (2, 58). Millet (foxtail millet, finger millet, sorghum) diet regulated the glucose level in the diabetic patients better than the non-millet diet (59). Breakfast consumption trial of Pearl millet porridge (PMP) and popular Scottish oats porridge (SOP) showed that responses of both were comparable, but PMP had a larger iAUC (incremental area under the curve) for gastric volume and a lower GIP (glucose-dependent insulinotropic polypeptide) responses compared to SOP (60).

### 4.2 Millets for cardiovascular diseases

Cardiovascular diseases are the leading cause of death globally as per WHO. Millets consist of higher sterols and pinacosanols, which prevent cholesterol synthesis. It has been demonstrated in animal study that sorghum fed hamsters have lower non HDL cholesterol (61). Yin et al. (62) reported the positive effect of millet bran oil (MBO) and refined millet bran oil (MRO) consumption on lipid metabolism in obese mice. MBO reduced lipid accumulation in the liver, brown and white fat hypertrophy and dyslipidemia. It also decreased hepatic lipid peroxidation, the plasma oxidative stress, and hepatic oxidative stress, and increased the abundance of some benign bacteria, including *Akkermansia* and *Prevotellaceae*. Phenolic extract of kodo millet was effective in inhibiting the oxidation of LDL cholesterol and liposome (63). Sorghum has a high level of fiber in the diet and it decreases cholesterol uptake, binding bile acids in the small intestine and preventing them from entering the blood-stream, which is helpful for the prevention of cardiovascular diseases such as atherosclerosis, and stroke (64). Results from different i*n vitro* and animal studies indicated that the lipidic and phenolic fractions from sorghum modulate parameters related to dyslipidemia and the risk of cardiovascular disease, which resulted from the action of phytosterols, policosanols and phenolic compounds. Studies have shown that grain sorghum lipids and the co-product, DDGS (Dried Distillers Grains with Solubles) were able to promote cardiovascular health by reducing both plasma low-density lipoprotein (LDL) and liver cholesterol levels at different dosage levels (65). Millets mitigate atherosclerotic cardiovascular disease risk by lowering insulin resistance, better glycemic control, lowering non-high-density lipoprotein (HDL) cholesterol and lowering BP (66, 67).

### 4.3 Anti-hypertensive properties of millets

High blood pressure is a major health problem of the current generation and is reaching epidemic proportions. It is a serious illness if left undetected and untreated. Dietary interventions are recommended to alleviate the issue. Studies support that, millets, being a good source of hypotensive components such as dietary fiber, protein, minerals, and other phytochemicals, are a promising diet for hypertensive individuals. Consumption of foxtail millet protein hydrolysates was found to ameliorate hypertension and alleviate related cardiovascular diseases in spontaneously hypertensive rats. Activity of serum ACE (Angiotensin I Converting Enzyme), a crucial enzyme in the process of hypertension, and angiotensin II levels were significantly lower in the treated rats (68). Finger millet ethanol extracts were also found to cause antihypertensive effects in rats (69) by controlling the renin-angiotensin system. *In silico* studies indicated that foxtail millet bran glutelin-2 peptide fractions are potential natural ACE inhibitors (70). An isolate of sorghum α-kafirins was reported to inhibit the activity of the angiotensin I converting enzyme (71) to reduce blood pressure. Significant reductions were observed in both systolic and diastolic blood pressure values with whole foxtail millets diet in mild hypertensive patients (72).

### 4.4 Millets for thyroid gland function and combating obesity

Manganese is essential for thyroid hormone homeostasis and sorghum is its rich source, which helps in proper regulation of the thyroid gland promoting weight loss through regulating fat metabolism (64). Sorghum grain starch has 1.2-fold higher amylose than other fine cereals, and this resistant starch is advantageous for obese and diabetic people. Sorghum rich in tannins was reported to reduce weight gain in animals (73, 74) attributed to formation of complexes with starch, which helps to lower caloric intake. Polymeric tannins from sorghum naturally modify starch by interacting strongly with amylose forming resistant starch, which cannot be digested in the small intestine and thus reaches the large intestine, delivering the health benefits of dietary fiber (75).

### 4.5 Effect of millets on gut microbiome

Integrating millet into the diet improves both gut health and overall nutrition. Dietary fiber component in millet comprises 15–20% and contains non-starchy polysaccharides, arabinoxylan, and β-glucan (76). Insoluble fibers, such as cellulose and hemicellulose, add bulk to the stool and aid in regular bowel movements (19). Soluble fibers, including beta-glucans, arabinoxylans, and pectins, have gel-forming properties and exert various physiological effects. They exhibited gel-forming properties by absorbing water and thus increasing food viscosity, inhibiting macronutrient absorption, lowering dyslipidemia and the postprandial glucose response. It could be metabolized by the gut microflora (*Bacteroides* and *Bifidobacterium*) during colonic fermentation to produce short-chain fatty acids (SCFAs). Soluble dietary fibers reduce bile acid absorption in the small intestine (ileum) and increase bile acid excretion, resulting in enhanced hepatic bile production utilizing intracellular cholesterol and lowering blood cholesterol levels. Insoluble dietary fibers, such as cellulose and hemicellulose, are more susceptible to colonic fermentation by the gut microbiota and have a beneficial effect on insulin sensitivity. Insoluble fibers remove toxins from the digestive system, improve the fecal volume and intestinal transit by enhancing water-holding capacity (77, 78). Prebiotic-resistant starch, which is resistant to enzymatic digestion, increases the proportion of beneficial microorganisms such as probiotic *Bifidobacterium*, *Lactobacillus*, and *Akkermansia* (79). Finger millet is high in microbiota-accessible dietary fiber, and implicated in the anti-diabetic and anti-obesity properties (2, 79).

**FIGURE 2:**
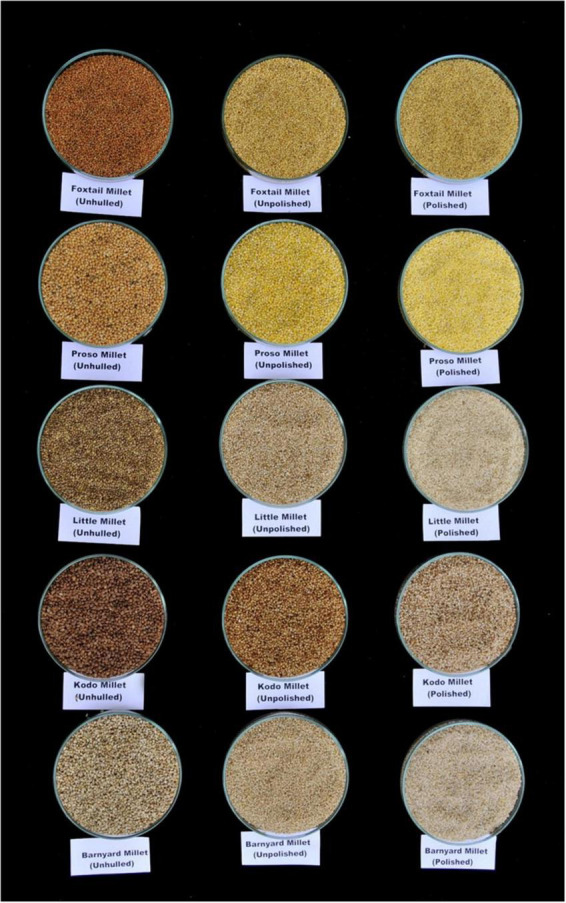
Hulled and unhulled grains of small millets.

The millet bran fraction has a significant amount of dietary fiber, that is not easily digestible. As a result, removing the bran portion during decortication/dehulling leads to a significant reduction in the fiber component (Figure 2). It is reported that dehulling millet grains over 30% leads to significant loss of nutritional fiber (80). Because most millets are consumed in their decorticated condition, it is critical to manage the degree of dehulling in order to optimize fiber content. Effect of milling on the fiber components of foxtail millet showed that the insoluble dietary fiber was low in the milled fraction than that of whole millet flour., whereas the fiber content of foxtail millet increased significantly with increasing germination time. (81, 82).

Digestibility of millet fibers varies with type of fiber, processing methods, and individual variations in gut microbiota. Processing alters the structure of fibers and impacts their digestibility and bioavailability. Fermentation process breaks down soluble fibers into smaller compounds, which are then absorbed by the colon cells and utilized for energy. However, the extent of fermentation and digestibility may differ between individuals due to variations in gut microbiota composition. Insoluble fibers can provide benefits by aiding in proper gut transit and promoting overall digestive health (83).

Dietary fibers can be prebiotic by nature if they are metabolized in the colon by the gut microbiota, to produce different SCFA such as acetic acid, propionic acid, and butyric acid (84). Gut microbiota hydrolyse non-digestible food fiber from millets into component monosaccharides in colonic fermentation using a unique set of enzymes not found in humans. These hydrolysed monosaccharides are then used by bacteria to produce various SCFAs *via* various routes. SCFAs reduce gut permeability from the gut lumen to colonic tissues and the liver (85), function as signaling molecules, to induce satiety, stimulate insulin secretion and lower glucose levels. SCFA-activated GPRs reduce colonic inflammation because butyric acid promotes mucosal healing by increasing epithelial cell migration (86, 87). SCFAs take part in metabolic activities, notably butyric acid, which is used by gut colonocyte cells to make the gut environment completely anaerobic, limiting the quantity and proliferation of potentially dangerous microorganisms like *E. coli* and *Salmonella* (88). Animal studies in acutely malnourished pigs showed that millet-based supplement restored the gut microbial diversity (89) and harmful bacteria, have decreased (90).

Consumption and subsequent fermentation of finger millet increased the abundance of *Pediococcus* and decreased the abundance of pathogenic bacteria (91). Furthermore, the production of SCFAs through millet fiber fermentation has been linked to reduced risk of metabolic disorders, such as obesity and T2DM (92). Regular consumption of millet fibers has been linked to improved gastrointestinal function, enhanced nutrient absorption, and reduced risk of various digestive disorders (93). High-fiber diets of millets are associated with a decreased risk of developing colorectal cancer (94). Foxtail millet consumption was found to lower colonic inflammation and the risk of AOM/DSS-induced colitis-associated CRC in rats. The regulatory effects were mediated by foxtail millet microbial metabolites activating aryl hydrocarbon receptor (AHR) and G-protein-coupled receptors (GPCR) and inhibiting STAT3 phosphorylation (95).

Millet is considered a good source of dietary fiber, with different types of millet varying in their fiber content. It is important to note that the recommended daily intake should be achieved through a balanced diet that includes a variety of fiber-rich foods, rather than relying solely on millet fibers. While many individuals can tolerate and benefit from increased fiber intake, some may experience gastrointestinal discomfort or changes in bowel movements when significantly increasing their fiber intake. It is advisable to gradually increase fiber intake over time and ensure adequate fluid consumption to help prevent potential gastrointestinal discomfort. Additionally, individuals with specific health conditions, such as irritable bowel syndrome (IBS) or inflammatory bowel disease (IBD), may need to consult with a healthcare professional for personalized guidance on fiber intake (96).

#### 4.5.1 Effect of millets on gut-brain axis induced satiety

Gut-brain axis is a bidirectional communication pathway connecting the gut and the brain. Emerging research suggests that the gut microbiota, which is influenced by dietary factors including fiber intake, plays a crucial role in modulating brain function and mental health. Millets, rich in dietary fiber, impact mood, cognition, and behavior. Gut microbiota communicate with the central nervous system through the immune system, the vagus nerve, and the production of neuroactive compounds. They produce a wide range of neurotransmitters, such as serotonin, dopamine, and gamma-aminobutyric acid (GABA), which can influence brain function and behavior (97). Butyrate produced by fermentation in the intestine can cross the blood-brain barrier and exert neuroprotective effects, promoting brain health and potentially influencing mood and behavior (98). Pre-clinical and clinical studies are exploring the therapeutic potential of dietary fiber interventions, including millet fibers, in conditions such as depression, anxiety, and neurodegenerative disorders. While the precise mechanisms underlying the effects of millet fibers on mental health are still being elucidated, the promotion of a diverse and balanced gut microbiota through fiber-rich diets is considered beneficial for overall brain health and mental well-being.

### 4.6 Effect of millets on colon health

Peptic ulcer disease also known as gastric ulcers, is a common disorder of the gastrointestinal system, causing gastric mucosal injuries due to an imbalance between the defensive and the aggressive factors affecting the mucous. Millet is reported to be protecting the gastric mucosa against ulceration. Foxtail millet has long been used to treat vacuity heat of the spleen and stomach, stomach reflux vomiting and reduced food intake with abdominal distention, in traditional Chinese medicine (99). Bound polyphenols of the inner shell from foxtail millet bran displayed anti-inflammatory effects in LPS-induced HT- 29 cells and in nude mice (100). Foxtail millet has antiulcer activity through suppressed levels of plasma and mucosal TBARS (Thiobarbituric acid reactive substances) and increased gastric non-protein sulfhydryl (NPSH) digestive enzyme activities (100). Foxtail millet protein reduced gastric ulcers in mouse models through a down-regulation of inflammatory cytokine expression in gastric tissue and improved oxidative status (101).

Pretreatment with millet diets significantly prevented gastric mucosal lesion development in ulcerated rats through the depletion of NPSH levels (102). Millet diets promote ulcer protection by decrease in ulcer index, TBARS values, and increased NPSH concentrations. Foxtail millet diet showed preventive and gastroprotective effects in experimental gastric mucosal lesions in rats protecting against the development of acute gastric mucosal injury, the anti-ulcer response and extensive antioxidant effect. Heat-treated foxtail millet could alleviate the NAFLD effects effectively through the reduction of hepatic total cholesterol and triglyceride contents and by reducing NAFLD-related gut microbiota dysbiosis (46).

### 4.7 Anticancer effects of millets

Cancer is a leading cause of death worldwide. Due to their antioxidant property, millets are considered to halt the degenerative process in the body and prevent carcinogenesis. Kodo millet phenolic extracts rich in ferulic and *p*-coumaric acids were effective in inhibiting hydroxyl and peroxyl radicals and the proliferation of HT-29 cells (human colorectal adenocarcinoma cell line), which was by 75–100% inhibited by the same (63). Foxtail millet extracts from two varieties Jingu28 and Jingu34, could potentially inhibit the growth of human breast (MDA) and liver cancer (HepG2) cells in culture (103). Foxtail millet bran-derived bound polyphenol (BPIS) showed apoptosis in human colorectal cancer HCT-116 cells and in HCT-116-bearing nude mice through enhanced ROS production (104). BPIS induced ROS generation in HCT-116 cells followed by ROS-triggered apoptosis. A peroxidase enzyme from foxtail millet bran was found to inhibit cell migration in human colon cancer cells through antagonizing epithelial-mesenchymal transition (EMT) *via* STAT3 signaling pathways (100). BPIS showed antiproliferative activity in the colon cancer cell line, HT-29, by upregulating miR-149 expression and inhibiting tumor aerobic glycolysis (105).

Bioactive compounds like vanillin extracted from proso and barnyard millets inhibited the cell proliferation and apoptosis induction in colon cancer cell lines (106, 107). Bran of certain sorghum lines (especially black sorghum) has up to 10 mg/g 3-deoxyanthocyanidins (108). This compound showed strong anti-proliferative activities when tested against various human cancer cell lines and its potential was comparable to that of quercetin, one of the most potent antioxidant molecules (109–111).

### 4.8 Millets in allerginicity treatment

Celiac is a disease that damages the small intestine and interferes with the absorption of nutrients from food. Grains like wheat, barley and rye contain gluten proteins, which cause an immunological reaction in susceptible individuals with celiac disease. This response results in the production of autoantibodies and the small intestine’s villi being destroyed causing nutritional malabsorption and other autoimmune illnesses. People from this disease cannot tolerate gluten. Millets are gluten-free and non-allergenic; a great grain for individuals with celiac disease and gluten sensitivity. *In vitro* and *in vivo* tests using sorghum-derived food products, did not cause any gastrointestinal or non-gastrointestinal symptoms demonstrating the safety of gluten free sorghum for patients with celiac disease (112). Immunochemical assays also proved the absence of toxic gliadin-like protein in sorghum and confirmed the safety of sorghum-based foods for celiac patients (113). Introduction of gluten-free breads and other baked products in the diet for celiac patients through millet intervention is recommended (114).

## 5 Other diseases

Overproduction or under-excretion of uric acid leads to the elevated serum uric acid, known as hyperuricemia, resulting in numerous diseases in humans. New drug-like molecules from millets as luteolin is a natural source to prevent and treat hyperuricemia and related diseases (115). Polyphenols have demonstrated potential anti-Alzheimer’s disease effects in cellular and animal studies. Complex mixtures of polyphenols in sorghum grain extracts, such as 3-deoxyanthocyanidins (DXA) have the potential to inhibit neurotoxic aggregation to prevent and treat Alzheimer’s disease (116). Millets have also demonstrated neuroprotective effects in Parkinson’s disease and other cognitive disorders (117).

## 6 Millets as functional foods

Functional foods offer health benefits that extend beyond their nutritional value and contain supplements or other additional ingredients. Millets are fortified with vitamins, minerals, probiotics and fiber. Besides primary nutrients, what makes millets therapeutic is the presence of phenolic compounds, which are the predominant form of secondary metabolites (Table 3). These are phytosterols, lignins, polyphenols, phytocyanins, and phytoestrogens. These compounds act as antioxidants thereby preventing damage to cellular membranes or genetic material within the cell. A number of health-promoting and protective properties have been attributed to millet phenolics and they were found to offer benefits like antimicrobial, immuno-modulatory, anti-inflammatory, antiviral, anticancer, antiplatelet aggregation, and inhibitory activities on cataract formation and digestive enzymes (118, 119). Polyphenols are found in the free as well as bound forms. Bound polyphenols have multiple biological activities including antioxidant, antitumor, immunomodulation, antifungal and anti-hyperglycemia effects (120). Hulls are richer sources of total phenolic contents (TPC) and dehulling was found to reduce the antioxidant properties of millet grains (121). Millet phenolics (40) protect oxidative DNA damage and hydroxyl radical-induced protein fragmentation, inhibit protein glycation, and reduce the formation of protein aggregates. Phenolic extracts of kodo and finger millet were highly effective (97%) against peroxyl-mediated DNA scission among millets (122). Grain (crude) extracts of kodo and finger millet are superior in TPC (total phenolic content) and TFC (Total flavonoid content) among millets and they inhibited collagen cross-linking and glycation (123). Chethan et al. (124) identified nine phenolic acids which included gallic acid, protocatechuic acid, p-hydroxybenzoic acid, vanillic acid, ferulic acid, syringic acid, trans-cinnamic acid, and p-coumaric acid in millets. On the other hand, Chandrasekara and Shahidi (125) found that hydroxycinnamic acids and their derivatives were the main contributors to the total phenolic compounds of insoluble-bound phenolic fraction of millet varieties. However, in another study by Xiang et al. (126), flavonoids were found to be the predominant phenolic compounds in different millet varieties, whereas Sharma et al. (127) reported higher amounts of phenolic content and antioxidant activity in methanolic extracts of kodo millet grains. It is, however, important to realize that different analysis methods also affect the total phenolic compound contents of plants.

**TABLE 3:** Important grain phytochemicals identified in millets.

S No	Crop	Name of the compound	References
1	Sorghum	(+)-Catechin, Cinnamaldehyde, 3-deoxyanthocyanidins, Stigmasterol, (−)Epicatechin, trans-Cinnamate, Luteolin, Sitosterol, Antheraxanthin, Apigenin, 5′-Prenyleriodictyol, Kaempferol-3-O-arabinoside, Genistein, Coniferyl aldehyde, Eriodictyol, Hesperetin, (−)-Epigallocatechin, 2-Coumarinate, 4-Coumarate, Lupeol, Geranylgeranyl diphosphate, Farnesyl diphosphate, Squalene, (+)-Neomenthol, glycitein, formononetin, ononin, and hispidulin, 8-Oxogeranial, Zeaxanthin, Citraconate, Carlactone, Spermidine, (−)-Epiafzelechin, 3-Dehydroshikimate, Naringin, Eriocitrin, Brassicasterol, 4-Coumaryl alcohol, Campesterol, Pentahydroxyflavanone, Kaempferol, Kaempferide, Caffeic aldehyde, p-Coumaraldehyde, Luteolin 7-glucoside, 2′,5-Dimethoxyflavone, Eriodictyol-7-O-glucoside, 2′,5-Dimethoxyflavone, Taxifolin, Galangin, Garbanzol, 5-Hydroxyconiferaldehyde, Chorismate, Caffeoyl-CoA	Ramalingam et al. (134)
2	Pearl millet	p-Hydroxybenzoic, protocatechuic, vanillic, Caffeic, p-coumaric, cinnamic, sinapic, trans -ferulic, Apigenin, myricetin	Chandrasekara and Shahidi (122), N’Dri et al. (143)
3	Barnyard Millet	2,3,4-Trihydroxybutyric acid, d-Ribose, Ribonic acid, Pentanedioic acid, Pentitol, D-Mannitol, alpha.Glycerophosphoric acid, Eicosanoic acid, Stigmasterol and beta.-Sitosterol Malic acid, Glutamine, 1,2,3-Propanetricarboxylic acid, Tetradecanoic acid, D-Mannose and Talose Butanedioic acid, 2,3,4-Trihydroxybutyric acid, Glutamine, L-Proline, Tetradecanoic acid, Hexadecanoic acid, alpha.-Glycerophosphoric acid, 9,12-Octadecadienoic acid, D-Mannose, D-Glucuronic acid, Eicosanoic acid, Docosanoic acid, D-Glucopyranose, Stigmasterol and beta.-Sitosterol	Padhiyar et al. (144)
4	Finger Millet	Gallic, p-hydroxybenzoic, protocatechuic, syringic, gentisic, vanillic, Caffeic, p-coumaric, cinnamic, sinapic, trans -ferulic, Catechin, gallocatechin, epicatechin, epigallocatechin, taxifolin, vitexin, tricin, luteolin, myricetin, quercetin, apigenin, kempherol, narigenin, diadzein, Procyanidin B1, Procyanidin B2,	Rao and Muralikrishna (145), Chethan et al. (124), Shobana et al. (119), Viswanath et al. (118), Chandrasekara and Shahidi (122), Banerjee et al. (146), Xiang et al. (126)
5	Foxtail	Gallic, p-hydroxybenzoic, protocatechuic, syringic, gentisic, vanillic, Caffeic, p-coumaric, sinapic, trans -ferulic, Catechin, quercetin, apigenin, kempherol, naringenin, isoscoparin glucoside, isorhamnetin glucoside, carthamidin diglucoside, orientin, deoxykievitol, luteolin, isogentisin, chrysoeriol, precarthamin, eupatorin, camelliaside B, diadzein	Chandrasekara and Shahidi (122), Pradeep and Guha (147), Wang et al., (148)
6	Proso millet	Ferulic acid, Sinapic acid, Chlorogenic acid, p-Coumaric acid, Syringic acid, Vanillic acid, 4-Hydroxybenzoic acid, Isoferulic acid, p-Hydroxycinnamic acid, Cryptochlorogenic acid, Neochlorogenic acid, Syringaldehyde, Sinapinaldehyde, p-Coumaraldehyde, Methyl ferulate, Caffeic acid, Ferulic acid, Sinapic acid, p-Coumaric acid, Syringic acid, Vanillic acid, 4-Hydroxybenzoic acid, Isoferulic acid, Syringaldehyde, Sinapinaldehyde, Protocatechuic acid-4-O-glucoside,, flavonols, Kaempferol 3-O-glucoside, Kaempferol 3-O-galactoside, Quercetin 4′-O-glucoside, Isorhamnetin 3-O-neohesperidoside, Flavanones, Naringenin O-malonylhexoside, Naringenin 7-O-glucoside, Naringenin, Hesperetin 5-O-glucoside, Hesperetin, Homoeriodictyol, 4′,5,7-Trihydroxyflavanone, Xanthohumol, Eriodictyol, Dihydrochrysin, Hesperidin, Isoflavones, 2′-Hydroxygenistein, Orobol, Genistin	Chandrasekara and Shahidi (122); (149)
7	Kodo millet	Gallic, p-hydroxybenzoic, protocatechuic, syringic, vanillic Chlorogenic, caffeic, p-coumaric, sinapic, trans -ferulic, cinnamic, Kempherol, apigenin, vitexin, isovitexin, luteolin, quercetin, Catechin, naringenin, taxifolin, Pterin-6-carboxylic acid, campesterol, methyl vanillate, arachidonic amide, N (3,5-dinitropyridin, 2yl) L-aspartic acid ester, N-propyl 9,12,15-octadecatrienoate, pregan-20-one, octadecenoic acid, hexadecanoic acid, methyl 10-trans, 12-cis octadecadienoate, stigmasterol, C-sitosterol, and pregnenolone	Chandrasekara and Shahidi (122), (150)
8	Little millet	Gallic, protocatechuic, syringic, gentisic, vanillic, Caffeic, p-coumaric, sinapic, trans -ferulic, Apigenin	Chandrasekara and Shahidi (122), Pradeep and Guha (147)

Grain sorghum whole kernel oil (phytosterol rich oil or policosanol rich wax) is found to serve as a possible heart health ingredient in functional foods (128). Jobelyn is a traditional herbal preparation based on *Sorghum bicolor* leaf sheath *SBLS* in west Africa and it is available online in the form of capsules. This standardized dried powder is a unique combination of phytochemicals that target the management of HIV-AIDS, chronic inflammatory conditions and anemia (129). Jobelyn is rich in carbohydrates, protein, dietary fiber, iron, natural vitamins like B12 and vitamin C. It also contains Selenium, Omega 3,6 and 9 and other essential elements and fatty acids.^1^

All these research points toward the immense potential that millets have to be explored as nutraceuticals. Still there is a need for more, systematic *in vitro*, and *in vivo* investigations to demonstrate the health-promoting effects of millets and identifying the bio-active molecules associated with these effects. There is scope for exploring plant parts other than grains for their phytochemical properties as dietary supplements while bioefficacy and efficient delivery systems need attention. Millets as a potential source of nutraceuticals is indeed a big leap toward our goal of ensuring nutritional security for all.

## 7 Future research directions

Research and reports on the therapeutic applications are available in foxtail millet and sorghum to a greater extent, while finger millet and pearl millet are moderately explored. Proso millet and barnyard millet are researched to a lesser extent on medicinal value. The other small millets like little millet, kodo millet, barnyard millet, brown top millet and fonio need more attention. Brown top and proso millet are rich in protein; kodo millet has more bioactive compounds. Breeding programs on increasing the productivity of millets as food selectively opt for high yield, but breeding programs aiming for improvement of specialty compounds coupled with good yield can be taken up for developing varieties with high nutrient content having good medicinal value in view of the alarming rise of disorders Millets exhibit great diversity for grain quality traits, a promise to breed for specific end-use products. For example, in sorghum, many germplasm lines and parents with high total phenolic content and antioxidant activity were identified (130) that can be used in the breeding program for targeting improvement of nutraceutical properties to support food processing and value addition efforts in sorghum. In order to get outstanding recombinants in segregating generations, the parents of the hybrids must be good general combiners for the characters to which improvement is sought. To obtain superior hybrids with high grain quality and high grain yield traits either of the parents should be a good combiner for quality and yield traits, or the parents should complement each other for combining ability of the traits. Breeding procedures, which can exploit both additive and non-additive gene actions like biparental mating will help in combining the desirable yield and quality traits in millets. In pearl millet, enormous genetic variability is available for iron and zinc (30–140 mg/kg Fe and 20–90 mg/kg Zn), which was used efficiently for developing high-yielding cultivars with high iron and zinc. The world’s first biofortified pearl millet variety, the Dhanashakti was developed by utilizing the intra-population variability within ICTP 8203, which is an early-maturing, large-seeded and high-yielding open-pollinated variety that has been under cultivation in India since 1990. Nutritional quality traits for Fe (42 ppm) and Zn (32 ppm) were included in the varietal promotion criteria of pearl millet, which is first of its kind in any of the food crop and the world too (131, 132). After Dhanashakti, the first wave of biofortified hybrids released were AHB 1200 and HHB 299 with 73–77 mg kg^–1^ Fe and 39–41 mg kg^–1^ Zn in 2018-19, and the second set of hybrids released during 2019-2020 were RHB 233, RHB 234, AHB 1269, HHB 311 with 83–91 mg kg^–1^ Fe and 39–46 mg kg^–1^ Zn. Improved little millet variety, CLMV1 for iron and zinc, improved finger millet varieties for iron, VR 929 (Vegavathi), for iron, zinc and calcium CFMV1 (Indravati) and CFMV2 are developed in millets with superior nutritional quality (133). Several bioactive compounds like quercetin, 3 deoxy anthocyanidins, kaempferol glycoside and flavonoids have beneficial effect on health. Identification of genotypes rich in these bioactive compounds and breeding them for satisfactory yield can be an important component of breeding programs. Multi-omics research on various agronomic and nutritional traits in millets still lags behind as these crops remained underexploited till recently. Multiple high throughput omics techniques (genomics, transcriptomics, proteomics, metabolomics) have enabled the identification of phytochemicals having therapeutic potential in millets and also their biosynthetic pathways. Targeted and untargeted metabolomics studies alone or in combination with transcriptomics have been reported in millets (134–136). There have been large-scale mGWAS (metabolite genome-wide association studies) to aid in breeding for targeted metabolites in foxtail millet (137) and pearl millet (138). Studies especially on the metabolite diversity in millets to establish their therapeutic potential need to be explored. Millet cultivation is particularly pertinent in this era of global climate change, where erratic weather patterns are frequently witnessed, as millets can grow well in semi-arid and dry regions, where water and resources are scarce and soil quality is poor. Small millets like proso, barnyard, kodo, and browntop are short duration crops that fit very well in the agroecosystems for inter cropping and intermittent cropping and hold a great opportunity for increasing the productivity per unit area of land. In this regard, millets offer the supreme combination of sustainability and better nutrition.

Many food products equivalent to rice, wheat and maize can be prepared from millets and technologies for different millet food products like ready-to-cook (RTC) and ready-to-eat (RTE), recipes of worldwide and commercial scale production systems are ready at many places including ICAR-Indian Institute of Millets Research, Hyderabad, India. Degree of decortication of the millet grains can be decided based on the end-use application, such as, for use in food products it can be decorticated to a higher degree, and for therapeutic purposes the degree of decortication can be reduced. Millets contain bioactive peptides that may be used to create functional food ingredients, however more investigation is needed to ascertain these peptides’ effects *in vivo* (139). Despite the existing body of research, several gaps in knowledge regarding the therapeutic properties of millets exist. There is a need for more *in vitro* and *in vivo* studies on the therapeutic potential of various nutrient components of millets and the specific mechanisms of action. Future research should include well-designed clinical trials and animal studies to address the gaps in current knowledge. Nutrigenomics of millets is yet to be explored, which would provide insights into the effect of nutritional components of millets on the health of individuals. Millet based food products for specific diets is a developing area opening enormous entrepreneurial opportunities.

## 8 Conclusion

Composition of millet grains, including their ample amounts of proteins, necessary amino acids, dietary fiber, vitamins, minerals, essential fatty acids, antioxidants, and other phytochemicals, makes them a valuable addition to the diet. Because of their hypoglycemic, anti-proliferative, anti-atherosclerogenic, antioxidant, anti-hypertensive, anti-inflammatory, and antimicrobial qualities, millet has been linked to improved human health. Benefits of millets in diet provide better nutrition by supplementing especially with minerals and vitamins that keep the individuals in good health and keep many disorders at bay. Presence of bioactive compounds reduces and slows the progress of lifestyle disorders and ailments most importantly by scavenging. Elimination of nutrient deficits like zinc and iron can be overcome with the help of millets. Leads at the research front indicate that many answers to today’s issues, from diabetes management to obesity and starvation, lie in millets. Millets are nutritional and hold a high promise for the therapeutic and pharmaceutical industry as nutraceuticals.

## Author contributions

JJ: Supervision, Visualization, Writing – original draft, Writing – review and editing. VK: Writing – original draft, Writing – review and editing. ChA: Writing – original draft, Writing – review and editing. MB: Writing – original draft, Data curation. CA: Writing – original draft, Writing – review and editing. KM: Writing – original draft. TN: Funding acquisition, Writing – review and editing. CS: Funding acquisition, Writing – review and editing, Resources. KV: Writing – review and editing, Conceptualization, Supervision, Visualization, Writing – original draft.

## Footnotes

1
https://clinicaltrials.gov/ct2/show/NCT02240173
